# Genotypic and Phenotypic Characterization of IncX3 Plasmid Carrying *bla*_NDM-7_ in *Escherichia coli* Sequence Type 167 Isolated From a Patient With Urinary Tract Infection

**DOI:** 10.3389/fmicb.2018.02468

**Published:** 2018-10-23

**Authors:** Yingying Hao, Chunhong Shao, Yuanyuan Bai, Yan Jin

**Affiliations:** Department of Clinical Laboratory, Shandong Provincial Hospital Affiliated to Shandong University, Jinan, China

**Keywords:** NDM-7, carbapenemase, *Escherichia coli*, multi-drug resistance, China

## Abstract

Infections due to New Delhi metallo-beta lactamase (NDM)-7-producing *Escherichia coli* are infrequent and sporadic. In this study, we report one case of recurrent urinary tract infection caused by *bla*_NDM-7_-producing *E. coli* belonging to phylogenetic group A, sequence type (ST) 167. In this study, we aimed to describe the genotype and phenotype of *bla*_NDM-7_-producing *E. coli* in China. The isolate exhibited resistance to β-lactam antimicrobials, trimethoprim-sulfamethoxazole, quinolones, and aminoglycosides. *bla*_NDM-7_ is located on a conjugative plasmid designated pJN05NDM-7 belonging to type IncX3. pJN05NDM-7 was fully sequenced and compared with all publicly available *bla*_NDM-7_-harboring plasmids. pJN05NDM-7 is almost identical to pKpN01-NDM7 and pKW53T, although the plasmids are geographically unrelated. The comparison of IncX3 plasmids harboring *bla*_NDM_ in China showed high similarity, with genetic differences within insertion fragments. Notably, the differences in plasmids of animal and human origin were insignificant, because only one plasmid showed deletion inside the ISAba125 region compared with pJN05NDM7. Our study demonstrates that *E. coli* carrying IncX3 plasmids play an important role as a reservoir and in the spread of *bla*_NDM_. Further studies should be performed to control the dissemination of *bla*_NDM_ among food animals.

## Introduction

New Delhi metallo-beta-lactamase (NDM)-producing bacteria are spread worldwide and pose a serious threat to public health, and is highly disseminated in China (Zhang et al., 2017; Liu et al., 2018). The surveillance for carbapenem-resistant *Enterobacteriaceae* (CRE) showed that *bla*_NDM_ production was the second major mechanism of carbapenem resistance *in Escherichia coli*, and *bla*_NDM-1_ was the most frequent variant (Khan et al., 2017). Since the first report on NDM-1 in 2009, 20 variants of NDM have been assigned in the Lahey Clinic database (Liu et al., 2018). NDM-7, which differs from NDM-1 by two point mutations corresponding to amino acid substitutions, was described in 2013 with increased carbapenemase activity compared with NDM-1 (Cuzon et al., 2013). *bla*_NDM-7_ is infrequently detected, and sporadic cases of infections due to *bla*_NDM-7_-producing enterobacteria have been reported in France, India, the United States of America, and Japan (Cuzon et al., 2013; Chen et al., 2015; Wang et al., 2016; Devanga Ragupathi et al., 2017; Pal et al., 2017; Sugawara et al., 2017; Espinal et al., 2018). In China, *bla*_NDM-7_-producing *E. coli* ST131 was first reported in 2016; however, the genetic content of *bla*_NDM-7_-harboring plasmids was not clearly described (Wang et al., 2016). Considering its global distribution, increasing attention should be paid to epidemiological survey of *bla*_NDM-7_.

In this study, we detected a *bla*_NDM-7_-producing *E. coli* isolate from a patient without a history of traveling admitted in a Chinese Hospital. To elucidate the molecular epidemiology and evolutionary dynamics involved in the dissemination of *bla*_NDM_, the genomic content and in-depth molecular characterization of the strains was determined in this study.

## Materials and Methods

### Bacterial Strains

The carbapenem-resistant *E. coli* strain JN05 was recovered from urine sample of a 61-year-old woman with recurrent urinary tract infection at a teaching hospital in Shandong Province of China in 2015. The patient was diagnosed with vesicovaginal fistula secondary to cervical cancer after chemotherapy and electrocautery surgery in 2009. According to the abdominal ultrasonography, the patient was diagnosed with hydronephrosis and hydroureter of upper segment on admission. After the treatment with multiple antibiotics failed, nephrostomy was performed to improve hydronephrosis. There was no history of traveling abroad. Informed consent was signed by the patient involved in this study. The methods in this study were approved by the Ethics Committee of Shandong Provincial Hospital and were carried out in accordance with the approved guidelines. The strain obtained from the patient was identified as *E. coli* by using Vitek-2 compact system and confirmed by Vitek-MS system (BioMérieux, France). Phenotypic detection of carbapenemases was performed using carbapenem inactivation method (CIM) and EDTA-modified CIM (eCIM) test.

### Antibiotic Susceptibility Assay

Susceptibility assay of antibiotics was performed on Mueller-Hinton (MH) agar plates using E test strips (Table 1). Susceptibility assay results were interpreted by Clinical Laboratory Standards Institute (CLSI) breakpoints (CLSI, 2017), with the exception of tigecycline, polymyxin B, and fosfomycin, which were interpreted by EUCAST breakpoints (EUCAST, 2017).

**Table 1:** Antibiotic susceptibilities of *Escherichia coli* JN05 and its transconjugant.

	Minimal inhibitory concentrations (μg/mL)																
Isolates	TZP	ATM	CZO	CRO	CAZ	FEP	FOX	IMP	MEM	ETP	AK	CN	CIP	LEV	SXT	FOS	TGC
JN05	> = 256	> = 256	> = 256	> = 256	> = 256	> = 256	> = 256	> = 32	> = 32	> = 32	> = 256	> = 256	> = 32	> = 32	> = 32	2	0.38
J05	> = 256	0.64	> = 256	> = 256	> = 256	16	> = 256	16	8	12	1	0.5	< = 0.02	< = 0.02	0.32	2	0.38
J53Azi^R^	< = 0.016	< = 0.016	< = 0.016	< = 0.016	< = 0.016	< = 0.016	< = 0.016	< = 0.02	< = 0.02	< = 0.02	< = 0.016	< = 0.016	< = 0.02	< = 0.02	< = 0.02	2	0.38


*TZP, piperacillin/tazobactam; ATM, aztreonam; CZO, cefazolin; CRO, cefatriaxone; FEP, cefepime; FOX,cefoxitin; IPM, imipenem; MEM, meropenem; ETP, ertapenem; AK, amikacin; CN,gentamicin; CIP, ciprfloxacin; LEV,levofloxacin; STX, trimethoprim/sulfamethoxazole; FOS, fosfomycin; and TGC, tigecycline.*

### Molecular Typing

Multilocus sequence typing (MLST) and phylogenetic typing was performed for molecular typing of the isolate as previous described (Wirth et al., 2006; Wang et al., 2016). The virulence factors of extraintestinal pathogenic *E. coli* (ExPEC)-associated genes were screened by PCR-based assays (Wang et al., 2016).

### Screening of Antibiotic Resistance Genes

Antimicrobial resistance genes were screened by PCR and DNA sequencing as described previously (Zhu et al., 2016). These antimicrobial resistance genes included carbapenemase-encoding genes, extended-spectrum β-lactamase genes, AmpC β-lactamase genes, 16S rRNA methylase genes, fosfomycin resistance genes, quinolone resistance genes, and polymyxin B resistance genes (mcr-1) (Du et al., 2016; Zhu et al., 2016).

### Analysis of *bla*_NDM_-Carrying Plasmids

Conjugation test was performed by mixed broth method using *E. coli* J53Azi^R^ as the recipient strain. Transconjugants were selected on MH agar plates containing 6 μg/mL ceftazidime and 100 μg/mL sodium azide. The antimicrobial susceptibility test of the transconjugant was carried out as antibiotic susceptibility assay of clinical strain.

The size and amounts of plasmids carried by the clinical isolate and transconjugant were evaluated by S1-pulsed-field gel electrophoresis (PFGE) as previously described (Liu et al., 2018).

### Plasmid Sequencing

The plasmid pJN05NDM carrying *bla*_NDM-7_ (present in strain JN05) was extracted and sequenced using an Illumina Hiseq platform and assembled by SOAPdenovo at the MajorBio Co (Shanghai, China). The gaps were closed through PCR and Sanger Sequencing at Sangon Biotech (Shanghai, China). The plasmid sequences were annotated by BLAST against the non-redundant protein database. PlasmidFinder was used for detection and typing of the plasmid.

## Results

### Resistance Profile of JN05 Strain

The carbapenem-resistant *E. coli* isolate JN05 was identified as metallo-beta-lactamase (MBL)-producing strains by eCIM. The JN05 strain was resistant to aztreonam, carbapenems, cephalosporins, quinolones, aminoglycosides, piperacillin-tazobactam, and trimethoprim-sulfamethoxazole, but was susceptible to fosfomycin, polymyxin B, and tigecycline (Table 1).

### Molecular Grouping, Resistance Genotyping, and Virulence Genotyping

The *E. coli* strain JN05 was assigned to ST167 and belonged to phylogenetic group A. It carried *papG* II, which may play an important role in the pathogenic process. Multiple antimicrobial resistance genes, including *bla*_NDM-7_, *bla*_CTX-M-3_, *bla*_CTX-M-14_, *bla*_TEM-1_, *qnrS, armA*, and *acc(6′)-Ib* genes, are responsible for the resistance profile of strain JN05.

### Analysis of the Plasmid Harboring NDM

New Delhi metallo-beta-lactamase -harboring plasmid of strain JN05 was successfully transferred into *E. coli* J53Azi^R^ by conjugation experiment. The presence of NDM-7 in the transconjugant was confirmed using PCR, and MLST was used to distinguish the transconjugants from the clinical strain. The transconjugant J05 was susceptible to aztreonam, quinolones, and aminoglycosides, but resistant to carbapenems and cephalosporin. S1-PFGE showed that the clinical strain JN05 harbored six plasmids, and the transconjugant J05 contained a single plasmid, which was approximately 46 Kb (Supplemental Figure S1).

pJN05NDM-7 is a 46,161-bp plasmid belonging to the IncX3 incompatibility group. The complete sequence of plasmid pJN05NDM-7 was submitted to GenBank under accession number MH523639. In pJN05NDM-7, *bla*_NDM-7_ was preceded by IS*3000*-IS*Aba125*-IS*5* in the upstream region and followed by *ble-trpF-dsbC*-IS*26*-Δ*umuD* in the downstream region. This *bla*_NDM_ genetic structure was common in *Enterobacteriaceae* for the horizontal transfer of *bla*_NDM_ (Pal et al., 2017).

**FIGURE 1:**
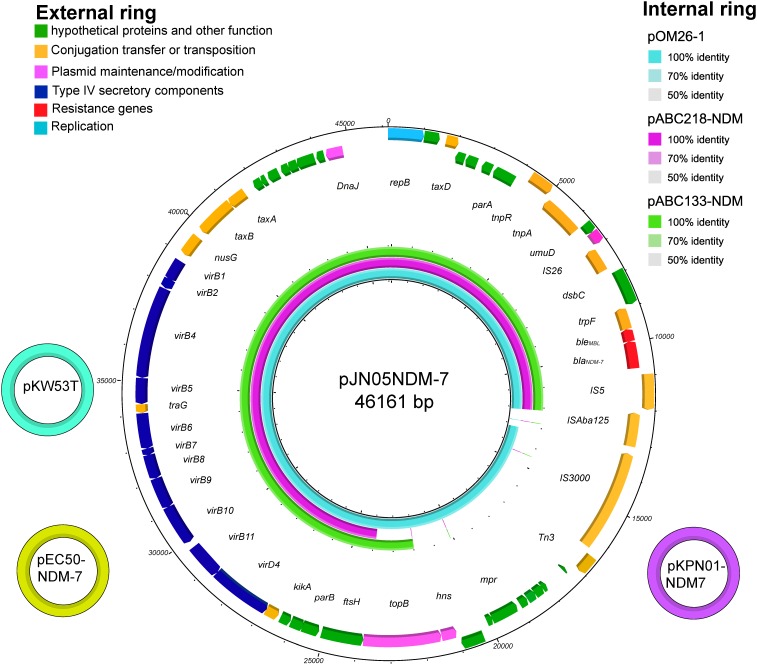
The three small external rings show different plasmids harboring *bla*_NDM-7_, shown in different colors that had >99% identity. The external ring represents the schematic map of plasmid pJN05NDM-7 (Accession No. MH523639). The genes were labeled with different colors according to their functional annotations. The internal three rings represent a comparative analysis of three *bla*_NDM_-harboring plasmids with pJN05NDM7, including pOM26 -1 (blue), pABC218 -NDM (purple) and pABC133 -NDM (green) (constructed by BRIG).

The full published sequences of seven plasmids harboring NDM-7 were downloaded and compared, including pKW53T-NDM (Accession No. KX214669), pEC50-NDM-7 (Accession No. KX470735), pKPN01-NDM-7 (Accession No. NZ_CP012990), pOM26-1 (Accession No. KP776609), pM110_X3 (Accession No. AP018141), pABC218-NDM (Accession No. KX214670), and pABC133-NDM (Accession No. KX214671) (Espedido et al., 2015; Pal et al., 2017). Sequence alignments revealed that pJN05NDM-7 was 100% identical to the previously described plasmid pKW53T-NDM of *E. coli* isolated in Kuwait (KW53T). pJN05NDM-7 plasmid showed high overall nucleotide identity (99%) with pEC50-NDM-7 from China and pKPN01-NDM-7 from Canada. In addition, pJN05NDM-7 was similar to the plasmid pOM26-1 isolated from Oman, and pABC218-NDM and pABC133-NDM from UAE. However, they lacked mobile genetic elements or even the topoisomerase III gene (Figure 1).

To explore the geographic distribution of IncX3 plasmids harboring *bla*_NDM_ in China, 29 plasmids were screened and analyzed, including two plasmids from North China (pEc1929, pNDM5-E6CN), 14 plasmids from East China (NUHL24835, p112298-NDM, pAD-19R, pNDM-5_IncX3, pNDM5-SSH006, pNDM-20, pNDM-QD28, pNDM-QD29, RJA274, pYE315203, pYQ13500-NDM, pZHDC33, pZHDC40, and pJN05NDM), six plasmids from South China (p112298-NDM, pCREC-A6-NDM, pNDM-HF727, pP785-NDM5, pP788A-NDM5, and pP855-NDM5), four plasmids from Central China (pEC50-NDM-7, pNDM-HN380, pP744T-NDM5, and pP768-NDM-5), and three plasmids from West China (pECNDM101, p3-NDM, and pSCE516-2) (Supplementary Figure S2).

Multiple NDM variants were harbored in the plasmids, including NDM-1, NDM-5, NDM-7, NDM-13, NDM-17, and NDM-20. We observed that the IncX3 plasmids carrying *bla*_NDM-5_ originating from different provinces of China showed high similarity, except three plasmids with various lengths of insertion sequences (pP744, pRJA274, and pZHDC40). Six IncX3 plasmids originating from pigs and one plasmid from chicken were identical to pNDM-HN380, thus confirming that this mobile NDM vector is widespread in China (He et al., 2017; Ho et al., 2018).

**FIGURE 2:**
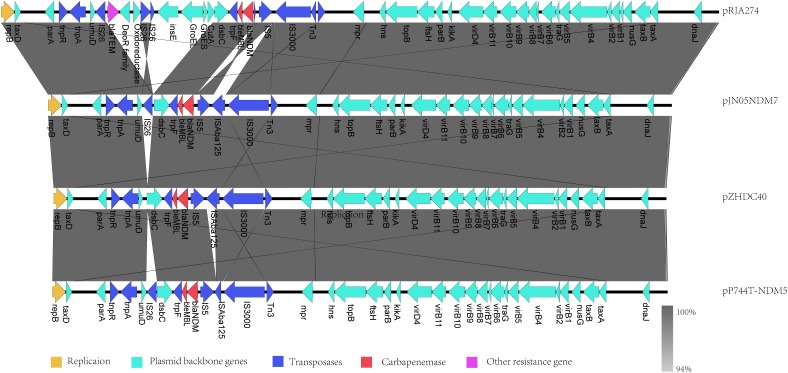
Comparison of the IncX3 plasmids harboring *bla*_NDM_ from China with pJN05NDM7.

As obvious differences were observed among sequences of pJN05NDM-7, pP744, pRJA274, and pZHDC40, linear structural comparison of whole genome sequences of pJN05NDM with the above plasmids was performed (Figure 2). The backbone of these plasmids showed high degrees of conservation and similarity, with sequence polymorphism at the region of additional insertion around the NDM gene. The plasmids did not carry any resistance genes other than NDM, except pRJA274. pRJA274 is a 53,134-bp circular IncX3 type plasmid haboring two resistance genes including *bla*_NDM-1_ and *bla*_SHV -12_. pRJA274 is almost identical to pJN05NDM-7, but the IS*Aba125* element (935-bp) between IS*3000* and IS*5* at the nucleotide position 40,753 was missing. In addition, the backbone of pRJA274 shared identity with plasmid pIncX-SHV. Compared with pJN05NDM-7, the inserted sequence of pP744 was different, with a deletion of 543 bp at IS*Aba125* located downstream of IS*5*. In pZHDC40, deletion of 816 bp at IS*26* downstream of NDM-7 was observed.

## Discussion

In this study, we aimed to evaluate the genotype and phenotype of *bla*_NDM-7_ -producing *E. coli* in China and found that *E. coli* carrying IncX3 plasmids play an important role as a reservoir and in the spread of *bla*_NDM_.

Although ST131 is the most prevalent strain type of *E. coli* worldwide, ST167 is considered to be related to clinical infections in China (Yang et al., 2014). In this study, JN05 assigned to ST167 was isolated from a 61-year-old woman with recurrent urinary tract infection. The isolate JN05 belonged to phylogroup A and was positive for *papG II*, which increased the ability of P-fimbriae adhesin (Wang et al., 2016). In addition, this isolate contained multiple resistance genes, including *bla*_TEM-1_,*bla*_CTX-M-3_, *bla*_CTX-M-14_, *bla*_TEM-1_, *qnrS, armA, and acc(6′)-Ib* genes; therefore, showed multidrug resistance and increased resistance to β-lactam drugs.

According to the surveillance of CRE strains in China, *bla*_NDM_ was mainly responsible for carbapenemase resistance in *E. coli*, while *bla*_NDM-7_ was relatively uncommon. Since the first report on clinical infection due to *bla*_NDM-7_ in France, this is the first report on fully sequenced plasmid carrying *bla*_NDM-7_ isolated from China.

*Escherichia coli* isolates carrying *bla*_NDM-7_ belonging to different STs were sporadic reported worldwide (Cuzon et al., 2013; Wang et al., 2016; Devanga Ragupathi et al., 2017; Pal et al., 2017; Espinal et al., 2018). According to previous reports, *bla*_NDM-7_ gene can be carried by several Enterobacter species and multiple types of plasmids including IncX3, IncF, and IncA/C groups, with sizes ranging from 37 to > 100 kb. IncX3, a self-conjugative plasmid, was most frequently observed to be the carrier of *bla*_NDM-7_.

Interestingly, the plasmid pJN05 was identical to the plasmid pKW53T-NDM-7 isolated in Kuwait. Geographical contiguity or travel history could not be considered as a cause of resistance gene transmission, suggesting that the plasmids maybe native and not imported. We proved that plasmids harboring *bla*_NDM_ were hidden in the environment and in the human gut worldwide long before we identified them. It is possible that IncX3 plasmids carrying different variants originated from the same plasmid, but point mutations during transmission and evolution generated the differences. Exposure to carbapenem agents speed up the evolution of plasmids carrying *bla*_NDM_ variants and enhance enzyme activity toward carbapenems.

Notably, *bla*_NDM_-producing isolates of animal origin increased, indicating that food animals have become the reservoir of *bla*_NDM_ (He et al., 2017; Kong et al., 2017). To understand the geographical distribution and gene polymorphism among the plasmids originating from different region, IncX3 plasmids carrying *bla*_NDM_ isolated from different areas were compared. Six plasmids harboring *bla*_NDM_ originating from pigs or chickens showed high similarity (> 99%) to those from patients. Because carbapenems were not approved for use in food animals in China, we assumed that the NDM-producing isolates were introduced to the farm via contaminated feed and water. The food animals contaminated by bacteria harboring *bla*_NDM_ accelerated the spread of resistance genes among healthy population. Nonetheless, more data are needed to explain the dissemination of *bla*_NDM_ among animals and humans.

## Conclusion

In conclusion, this study identified self-transmissible IncX3 plasmids carrying *bla*_NDM_, which were disseminated in geographically segregated areas in China and other countries in the world. This study emphasizes the important role of IncX3 plasmids in transmission of *bla*_NDM_ in China. Effective measures should be taken to monitor and control the rapid dissemination of *bla*_NDM_.

## Author Contributions

YH and YJ contributed to experiment conception, design, and wrote the paper. CS and YB performed data analysis.

## Conflict of Interest Statement

The authors declare that the research was conducted in the absence of any commercial or financial relationships that could be construed as a potential conflict of interest.

## Supplementary Material

FIGURE S1 S1-pulsed-field gel electrophoresis (S1-PFGE) patterns of clinical isolate JN05. **(A)** PFGE of clinical isolate JN05; **(B)** PFGE of transconjugant J05; M, marker, Salmonella enterica serotype Braenderup H9812.

FIGURE S2 Alignment of pJN05NDM7 with 28 IncX3-typed plasmids harboring *bla*_NDM_ in China identfied in GenBank (To April 2018).
