# Supplementary figures and images for: Genotypic and Phenotypic Characterization of IncX3 Plasmid Carrying blaNDM-7 in Escherichia coli Sequence Type 167 Isolated From a Patient With Urinary Tract Infection
Source: Front Microbiol. 2018 Oct 23;9:2468. doi: 10.3389/fmicb.2018.02468 (PMC6205962; doi:10.3389/fmicb.2018.02468)

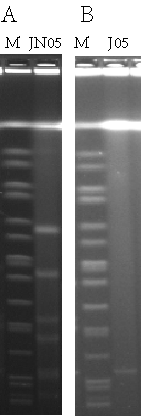

Supplement: FIGURE S1 — S1-pulsed-field gel electrophoresis (S1-PFGE) patterns of clinical isolate JN05. (A) PFGE of clinical isolate JN05; (B) PFGE of transconjugant J05; M, marker, Salmonella enterica serotype Braenderup H9812. [file Image_1.TIF]
